# Stroke-Induced Central Pain: Overview of the Mechanisms, Management, and Emerging Targets of Central Post-Stroke Pain

**DOI:** 10.3390/ph16081103

**Published:** 2023-08-04

**Authors:** Anugeetha Thacheril Mohanan, Sermugapandian Nithya, Yousra Nomier, Dalin A. Hassan, Abdulmajeed M. Jali, Marwa Qadri, Shamna Machanchery

**Affiliations:** 1 Department of Pharmacology and Toxicology, College of Pharmacy, Jazan University, P.O. Box 114, Jazan 45142, Saudi Arabia; 2 Department of Pharmacology, Sri Ramachandra Faculty of Pharmacy, Sri Ramachandra Institute of Higher Education and Research (Deemed to be University), Porur, Chennai 600116, Tamilnadu, India; 3 Inflammation Pharmacology and Drug Discovery Unit, Medical Research Center (MRC), Jazan University, P.O. Box 114, Jazan 45142, Saudi Arabia; 4 Department of Clinical Pharmacy, College of Pharmacy, Jazan University, P.O. Box 114, Jazan 45142, Saudi Arabia

**Keywords:** stroke, neuropathic pain, central post-stroke pain (CPSP), dysesthesia, somatosensory deficits, disinhibition, neuronal hyperexcitability, spinothalamic dysfunction, pharmacotherapy

## Abstract

The incidence of stroke plays the foremost role in the genesis of central neuropathic pain. Central post-stroke pain (CPSP) is a central pain arising from a vascular lesion in the central nervous system that elicits somatosensory deficits, often contralateral to stroke lesions. It is expressed as continuous or intermittent pain accompanied by sensory abnormalities like dysesthesia and allodynia. CPSP remains de-emphasized due to the variation in onset and diversity in symptoms, besides the difficulty of distinguishing it from other post-stroke pains, often referred to as a diagnosis of exclusion. Spinothalamic dysfunction, disinhibition of the medial thalamus, and neuronal hyperexcitability combined with deafferentation in thalamocortical regions are the mechanisms underlying central pain, which play a significant role in the pathogenesis of CPSP. The treatment regimen for CPSP seems to be perplexed in nature; however, based on available studies, amitriptyline and lamotrigine are denoted as first-line medications and non-pharmacological choices may be accounted for cases intractable to pharmacotherapy. This review attempts to provide an overview of the mechanisms, existing management approaches, and emerging targets of CPSP. A profound understanding of CPSP aids in optimizing the quality of life among stroke sufferers and facilitates further research to develop newer therapeutic agents for managing CPSP.

## 1. Introduction

A nerve injury or dysfunction in the central nervous system (CNS) may evolve into central neuropathic pain [1]. Neuropathic pain can result from CNS disorders due to vascular (ischemic or hemorrhagic) or traumatic events affecting the brain or spinal cord. Central pain is observed among subjects reported with stroke, spinal cord injury, and multiple sclerosis. Stroke is reported to be the second prime cause of death worldwide, and morbidity continues to leave the quality of subjects’ lives compromised. As stroke has a higher incidence among these diseases, it poses to be the root of central pain in stroke survivors [2] and plays a dominating role in central pain originating from the brain [3]. Pain after stroke is complex and is clinically classified as central post-stroke pain (CPSP), hemiplegic shoulder pain, pain due to spasticity, complex regional pain syndrome, and headache. CPSP is neuropathic in origin, producing insistent pain, with damage in the thalamus present among a significant proportion of stroke patients. In the 19th century, the central pain commonly occurring after thalamic strokes was described as ‘thalamic pain’. Later, it was recognized that lesions affecting extra thalamic regions or any part of the sensory tract may contribute to similar central pain. Thus, the phenomenon was widely perceived as CPSP. Apart from CPSP, the remaining post-stroke pain can be attributed to peripheral mechanisms; in a large percentage of patients, it was reported to be a combination of more than one subtype of post-stroke pain. The common combinations are found to be CPSP and spasticity pain, as well as CPSP and shoulder pain [4]. In general, one-third of post-stroke cases are disclosed with CPSP [5]. The onset period of developing CPSP is found to be higher at sub-acute and chronic stages rather than acute stages after stroke. The delay in the appearance of symptoms and distinct motor deficits post-stroke leaves it less attended. Additionally, the poor localization of pain and diverse symptoms make the diagnosis of CPSP difficult, and it is often identified by ruling out other potential causes. The persistent pain in CPSP impairs psychological well-being by disturbing normal sleep patterns as well as by causing a decline in social interaction, in addition to interfering with daily activities. Furthermore, leading a life with chronic pain and cognitive decline often leads to depression among stroke victims [6,7].

The complexity in distinguishing CPSP from other peripheral pain arising post-stroke de-emphasizes it among post-stroke pains. Though management of CPSP seems challenging, the use of appropriate diagnostic methods and analyzing the right therapeutic methods depending on the state of the patient may help in achieving progress in the state of pain [4]. There are several reviews on pharmacological management and assessment of the clinical efficacy of various therapeutic agents used in CPSP. The literature concerning the pathophysiology of CPSP is finite, as well. Enhanced awareness of the physiology of pain signaling, the mechanisms of central pain, and the consequences of stroke on the pain pathway may steer us toward an early diagnosis. The design of newer progressive interventions may modify the functional capability among sufferers. Hence, the present review aims to provide insights into the pain transmission and central pain mechanisms that may play a role in the pathophysiology of CPSP. Additionally, it focuses on the proposed diagnostic measures of CPSP, along with its pharmacological and non-pharmacological management regimes. Though the available pharmacotherapy for CPSP is limited, it is necessary to discover new therapeutic targets that can manage pain conditions more efficiently with fewer side effects. Thus, this review also compiles the emerging targets of CPSP that may guide and nurture future research in identifying new therapeutic agents for the management of CPSP. To provide an overview of CPSP, a literature search was carried out, inserting keywords like CPSP, post-stroke pain, central pain, pain pathways, pathophysiology of CPSP, central disinhibition, and management of CPSP. Relevant data were recovered to illustrate basic information as well as advances in CPSP and research defining central pain mechanisms.

## 2. Central Post-Stroke Pain (CPSP)

Central post-stroke pain (CPSP) is a neuropathic pain originating from a lesion in the CNS, which occurs as an impact of cerebrovascular diseases like stroke [8]. It may result in somatosensory deficits in correlation with ischemic or hemorrhagic lesions damaging the CNS spinothalamic pathways, which are often contralateral to the stroke lesions [9]. To be precise, the International Association for the Study of Pain has defined CPSP as ‘the pain originating as a direct consequence of a lesion or disease that may affect the central somatosensory functions’. Though CPSP may arise from an ischemic or hemorrhagic stroke, reports claim that subjects who encountered ischemic stroke had a higher incidence (86.1%) than hemorrhagic stroke (13.9%) [10]. The onset of central pain appears within a month or may protract up to 1–6 years following a stroke [11]; nevertheless, it is commonly observed within 1–3 months after a stroke [6]. The fact was supported by the Bashir et al. study, where the onset of pain was initiated at 3 months among 50% of subjects [12], hence justifiying the term ‘CPSP’, as the pain develops after a period of stroke occurrence. Pain symptoms in CPSP patients are mostly presented with dysesthesia, allodynia, and hyperalgesia [6], where the pain cannot be delimited to certain areas and may vary with days. Pain distribution is influenced by the location of sensory-motor defects as well [11].

## 3. Epidemiology and Risk Factors

Prevalence of CPSP after stroke exhibits a varying nature and is estimated as 1 to 35% [13], given that the differences in the selection criteria and unavailability of biomarkers for differentiating this pain category from concomitantly presenting other pain syndromes [14]. Apart from that the population included a heterogeneity of the lesions, difference in the time of assessment following stroke and variations in the onset of pain [15]. There has been a growing interest in stroke management measures; however, it is surprising to find very few epidemiological studies conducted to identify CPSP sufferers, despite being a primary complication observed post-stroke. The controlled cross-sectional study comparing the relationship of neuropathic symptoms cluster to quantitative sensory testing (QST) parameters in CPSP reported a high prevalence rate of 38.2%, whose assessment was done 47.7 months after stroke. The QST findings in CPSP assist in the diagnosis of CPSP and distinguish neuropathic pain from other PSPs in future trials [10]. A hospital-based prospective study conducted on 319 stroke patients on a follow-up visit found the CPSP prevalence to be 20.7% involving both thalamic and extra-thalamic regions, with 57.6% of lesions noted on the right side [16]. Another prospective study states that 5.9% of 824 patients from the Helsinki Young Stroke Registry with first-ever ischemic stroke in less than 50 years demonstrated CPSP at 8.5 years after stroke [17]. The onset of CPSP after the stroke substantially differs among the studies reported. Various cohort studies reported that most patients began experiencing the symptoms one to three months after the stroke [12,16,18]. In contrast, Ajit Kumar et al. also disclosed cases where CPSP was observed later than one year. Young age, location of the stroke lesions, and smoking may be considered as the risk factors from the studies conducted previously. A study conducted in the Helsinki Young Stroke Register found that young stroke patients are at risk of developing CPSP if there is a presence of large ischemic lesions with hemorrhagic transformation and co-relating CPSP with the severity of stroke with hemorrhagic transformation. However, another study reported that the occurrence of stroke lesions in the thalamic, cortical, and brainstem showed CPSP recurrently, whereas CPSP was rare with stroke confining to basal ganglia [18,19]. Osama Ahmad et al. demonstrated in their study that smokers (30.4%) are more likely to present CPSP than non-smokers (7.1%), with smoking considered a triggering factor.

## 4. Clinical Features and Diagnosis

Pain symptoms initiate with dysesthesia that might be spontaneous or evoked [4] and which accounts for nearly 85% of patients. Patients often experience spontaneous ongoing pain comprising of burning, pricking, freezing, aching, and a squeezing kind of pain presented alone or in combination [20] that may be triggered by various stimuli like touch, movement, stress, or cold [12]. Intermittent pain is often expressed as shooting and lacerating, making CPSP troublesome and leaving the quality of life compromised for those who underwent stroke [20]. Evoked pain finds its origin in nociceptive or non-nociceptive stimuli that include hyperalgesia, dysesthesia, and allodynia, commonly reported within CPSP patients. Allodynic scores were positively presented in 33–83% of CPSP patients elicited by a thermal (cold) or mechanical stimulus (touch). The absence of allodynic scores in pain-free stroke patients affected with somatosensory dysfunction ascertains the presence of allodynia and hyperalgesia in CPSP syndrome [21]. A study conducted by Barbosa et al. [10] identified 39 patients with central neuropathic pain after stroke; 79.5% of them presented pain located in the upper limbs, face, and lower limbs. The foremost symptoms observed included burning sensations in 82.1%, tingling in 66.7%, and pain evoked by cold stimulus in 64%. Moreover, hyperpathia accounted for 71.8% of CPSP patients that appeared frequently. Sensory abnormalities like thermal and mechanical allodynia also had a prevalence of 61.5%. Spasticity was reported for 53.8%, whereas upper limb disability was common among CPSP patients with cold hypoesthesia often visible in the face and both limbs. Bashir and colleagues [12] emphasized a burning sensation and a followed electric shock-like sensation in 62.5% and 25% of patients, respectively, as primary signs in a study conducted on CPSP patients previously. Abnormal pain descriptors like pins and needles were reported to be 44.4% and 33.3% among the participants. Furthermore, sensory abnormalities, hyperalgesia, and allodynia were reported to be 35.7%. Generally, the pain was noticed in the body regions confined with somatosensory abnormalities corresponding to the lesions present in the CNS [10]. The location of lesions can also be correlated with body regions presented with impairment at clinical investigation. Lesions at the thalamus mostly described symptoms of the hemi body contralaterally. On the other hand, when the lesions are localized at medullary regions, symptoms are specific to the location, if medial or lateral. The impairments are restricted to extremities and trunk, in case of medial lesions with numbness and tingling expressed as pain descriptors. On the contrary, lateral lesions expressed a facial disability that is either contralateral or ipsilateral with descriptors such as cold or burning. Alternatively, if the lesions are at the lenticular capsule, a higher incidence of pain symptoms is observed in the lower limbs compared to the upper limbs and face [22].

Diagnosis of CPSP probably presents a demanding task due to varying clinical symptoms and coexisting poststroke pains. As clear-cut diagnosis of CPSP is difficult, it may be based on integrating the patient’s history along with clinical examination, sensory tests, and neuroimaging of lesions in the CNS. CPSP is indicated contralateral to the affected cerebral hemisphere as stated by the International Association for the Study of Pain (IASP) guidelines. Somatosensory examinations will also serve as a tool in the prognosis of CPSP. The appearance of dysesthesia or allodynia and the absence or decreased sensation of a needle or cold stimulus can also augment the possibility of central pain after the stroke [15,16,17,18,19,20]. Several criteria have been proposed by researchers to diagnose CPSP among stroke patients in Table 1 [19,23,24].

## 5. Mechanisms and Pathophysiology of CPSP

### 5.1. Route of Pain Transmission

The pathway of pain involves various sites of the CNS, like the cerebral cortex, midbrain, and spinal cord. Pain inputs sent by nociceptors are received by the dorsal horn (DH) of the spinal cord [25] and are transmitted by the spinothalamic tract emerging from the lateral portion of the spinal cord, channeling to the ventral posterolateral nucleus (VPL) of the thalamus, and finally ceasing at cortical regions of the brain. Pain conduction from the brainstem bifurcates at the thalamus into the lateral and medial systems to reach the different cortical regions. In the lateral thalamocortical pathway, the sensory ventral caudal nucleus, on receiving sensory signals from the spinothalamic tract, directs it to the primary somatosensory cortex concerning pain discrimination. This nucleus also extends projection neurons to the secondary somatosensory cortex connected to the intensity of pain [13,20]. On the other side, the sensory tract converging with medial and intralaminar thalamic nuclei projects the noxious stimuli to the cingulate cortex involved in the affective component of pain, thereby comprising the medial thalamocortical pathway. Projection neurons connecting the insular and cingulate cortices are supported by their linkage to the parabrachial nucleus and amygdala [22,25]. Functional imaging studies show that the posterior insular cortex and medial operculum located in the cerebral cortex regions are thought to process the temperature and pain signals emerging from the spinothalamic tract [26]. When there are abnormalities between lateral and medial pain systems, this can lead to allodynia, where the pain is felt from a normal, non-painful stimulus. Pieces of evidence show that allodynia could be caused due to a diminished response of the cingulate, anterior cortex at the medial spinothalamicus and an intensifying insular activity in the lateral spinothalamicus [13].

The periaqueductal gray (PAG), situated in the midbrain, plays an integral role in pain transmission as a receiver point for ascending feed from the dorsal horn as well as incorporating the inputs from higher centers like the hypothalamus, amygdala, and frontal lobe. The descending circuit in the relay of pain access PAG is connected to the dorsolateral pontine tegmentum (DLPT) and rostral ventral medulla (RVM) via projection neurons for regulating the output from the spinal cord. Marked presence of neurotransmitters like noradrenaline (NA) and 5-hydroxy tryptamine(5-HT) have been identified in the PAG/RVM pathways apart from the endogenous opioid system [25].

### 5.2. Mechanisms Involved in Central Pain and CPSP

Injury to CNS in the form of vascular lesions, trauma, or infections leads to nerve impairment or damage in nerves of the somatosensory system and results in central neuropathic pain. In general, the proper transmission of pain signals to CNS is the balance of inhibitory and facilitatory influence in coordination with sensory inputs between the brain stem nuclei (PAG, RVM) and spinothalamic tract along with thalamocortical circuits. Any disturbance to this balance can be put forward as mechanisms underlying the central pain.

**Figure 1:**
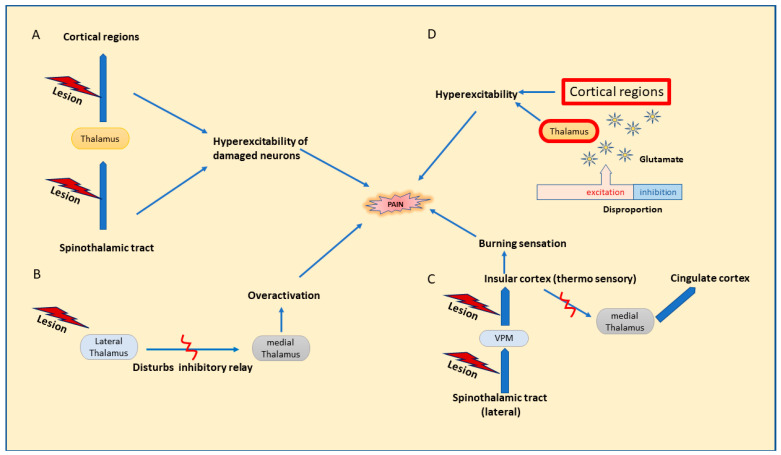
Possible mechanisms for central pain. (**A**). Dysfunction of the spinothalamic tract. (**B**). Disinhibition theory. (**C**). Thermosensory disinhibition theory. (**D**). Central sensitization.

Abnormalities in the central pain pathway have been denoted by the dysfunction of the spinothalamic tract, which is an integral part of the sensory pathway for somatic pain and thermal and touch sensations. Damaged nerves in the spinothalamic tract initiate complex reactions accompanied by neuronal hyperexcitability, eventually leading to hypersensitivity to cold and pinprick stimuli, which are common findings among CPSP patients rather than stroke patients without central pain [20]. Hypersensitivity can be explained as arising from the denervation of existing neurons of the spinothalamic tract post-injury [2]. Pain sensations are presented by a loss of inhibitory control within the neo spinothalamic tract (lateral spinothalamic tract) projections, which are fast-conducting and accelerating disinhibitions in the paleo spinoreticulothalamic (medial spinothalamic tract) projections [20]. A study demonstrated by Vartianien et al. corroborates the concept of the development of central pain implicated with damage in the spinothalamic cortical pathway (Figure 1A) and validated with the results from previous research stating CPSP as emerging from any defect concerning this pathway [27].

Disinhibition theory, proposed by Head and Holmes in 1911 [28], states that any lesion affecting the lateral thalamus hinders the inhibitory relay between the lateral and medial thalamus, leading to the overactivation of neurons in the medial thalamus, which results in increased pain perception [13,26,29], Figure 1B. Later, on revising the above hypothesis, thermosensory disinhibition theory states that the lesion in the lateral spinothalamic cortical projection neurons that connects to the insular cortex through the posterior ventral medial nucleus further results in the disinhibition of medial thalamic projections to cingular cortical regions [20]. Consequently, it may interfere with the cold signaling pathway in the lateral spinothalamicus and end in a burning sensation and increased responses to cold temperature; Figure 1C [6,15].

Disproportion in the excitation inhibition facilitates a rise in neuronal excitability in the thalamus or cortex due to increased binding of glutamate with NMDA receptors, leading to spontaneous pain due to a rise in intracellular calcium, constituting the central sensitization; Figure 1D [15]. Despite the augmented activity in the regions of pain sensations in the brain, central sensitization is related to altered processing of sensory inputs in the brain, ensuing in augmented neuronal signaling in CNS and evoking hypersensitivity to pain. Recently, central sensitization was redefined by IASP in 2017 to a broader term ‘nociplastic pain’ defined as ‘pain arising from altered nociception despite no clear evidence of actual or threatened tissue damage causing the activation peripheral nociceptors or evidence for disease or lesion of somatosensory system causing the pain’. It is expressed in the form of hypersensitivity arising post changes in pain sensory pathways involving CNS, suggestive of dysregulation in the pain pathways in CNS synonymously with central sensitization. It is observed as a comorbid condition with chronic pain, generally neuropathic or nociceptive in nature [30]. Hyperexcitability or an increased firing of the destructed afferent neurons in thalamocortical regions could be linked to spontaneous pain in CPSP [20]. To line up, Gritsch and his team, in a mouse study, suggested that the hyperexcitability observed in the lateral thalamus finds its relation connected to dysfunction in the inhibitory control of GABA and voltage-dependent calcium channel expression and leads to central pain [15]. Additionally, therapeutic agents mostly prescribed for treating central pain include drugs that partly subside neuronal hyperexcitability, which may back up the stated mechanism as well [20]. The onset of spontaneous pain in CPSP also owes mechanisms linked to brain plasticity changes that are associated with the hyperexcitability of neurons profound in thalamocortical regions [15].

Pain transmission is influenced by the neurotransmitters in the central nervous system and facilitated in deriving the theories behind central neuropathic pain. A balance between excitatory and inhibitory neurotransmitters in the neuronal tracts of the somatosensory system determines the proper transmission of pain. Neurotransmitters that are inhibitory in action, like glycine, GABA (Gamma amino butyric acid) along with monoamines (NA, 5-HT), and endogenous opioids, are found to inhibit the spinothalamic tract. Hyperexcitability of neurons arises from the release of the excitatory neurotransmitter glutamate at the site of nerve injury [3]. Interestingly, brain imaging studies confirm the increase in glutamate concentration during painful conditions [2]. The sodium and calcium channels are influenced by peptide hormones like cholecystokinin (CCK) and substance P (SP) at the spinal cord level, while potassium channels play an important role in regulating neuronal excitability by maintaining the resting membrane potential. Neuropeptides are also involved in the mechanism of pain at different levels. The calcitonin gene-related peptide (CGRP) found in the dorsal horn is released in the central and peripheral nervous system, facilitating calcium release and leading to excitation. SP, neurokinin A, and neurokinin B acting on G-protein coupled receptors like neurokinin type 1 (NK 1), neurokinin type 1 (NK 2), and neurokinin type 1 (NK 3), respectively, induce an excitatory effect. Prostaglandins also enhance chemical mediators like SP, CGRP, serotonin, and bradykinin, complementing pain sensitization [25].

### 5.3. Pathophysiology of CPSP

Knowledge regarding the pathophysiology of this neuropathic pain arising post-stroke is limited, though advances in functional imaging studies of the brain and factors that precipitate central pain have led to the perception of several aspects that may lead to the onset of CPSP. The thalamus is believed to play a significant role in mechanisms contributing to central pain and CPSP. Recent studies reveal the presence of lesions in the ventral posterolateral nucleus (VPL) or ventral posterior medial nucleus (VPM) of the thalamus among CPSP patients. A study conducted using modern techniques like magnetic resonance imaging (MRI) and digital radiographic atlases in thalamic stroke patients with CPSP found the presence of lesions in the VPL nucleus, specifically posterolateral and inferior parts, to a greater extent, unlike the smaller presence in the VPM nucleus [26]. At the same time, lesions in the pulvinar nucleus and VPN were found in a few patients. The findings comply with an earlier study, which demonstrated that thalamic infarcts confining to VPL and VPN caused CPSP on analyzing the thalamic infarcts in a series of patients [20], while Kim JH et al. also proposed the association of CPSP impeding the cold sensitivity with thalamic lesions in the ventral caudal nucleus [20]. In addition, interruption in afferent nerve fibers seen as a sequence after stroke may lead to VPL inhibition with a combined effect of central disinhibition due to loss of GABAergic/glycinergic interneurons, which subsequently leads to allodynia, characteristic pain in CPSP sufferers [31].

Furthermore, the study of Treister and colleagues states that the probability of CPSP is 81 times more if the thalamic lesions happen to be in the meeting point of ventral posterior nuclei and pulvinar nucleus [26]. As symptoms manifest after several weeks, brain plasticity may also indicate the emergence of CPSP [11]. Some areas in the cerebral cortex have been involved in CPSP. An investigation conducted on 24 patients disclosed the association of CPSP with ischemic injury to the insular cortex and operculum [26].

Convincingly, CPSP patients on magnetic resonance imaging (MRI) studies revealed the presence of distinctive patterns of cortical atrophy in various cortical regions like, insular, secondary somatosensory, ventrolateral prefrontal, including nucleus accumbens. Sensory deficits and abnormalities in affective pain may be explained by the anatomical changes observed within these regions [32]. Furthermore, studies utilizing diffusion tensor imaging put forward the findings on pain processing regions that include the thalamus, somatosensory cortex, and cingular and insular cortical regions that exhibit structural alteration in their white matter [33]. The findings in CPSP patients propose the physiological condition to find an association with the area of neuroanatomical damage and may guide the diagnostic criteria and therapeutic screening [34].

CPSP can be described and agreed on with ‘disinhibition theory’ and was confirmed with a study conducted in hyperpathic CPSP patients using single photon emission computerized tomography (SPECT), which presented thalamus hyperactivity when a pain stimulus was given on the hemiparetic side. Interestingly, this effect was not retrieved in the other three patients with the absence of hyperpathia. So, CPSP can arise from lesions in the thalamus, mostly the lateral thalamus [13,22]. Pain can be processed when there is an altered neuronal circuit with uninjured parts in the sensory pathway, as explained by disinhibition theory. Additionally, research using diffusion tensor tractography discloses that the lesion that involved the spinothalamic tract partially is more prone to develop CPSP than the patients with lesions with complete involvement of the spinothalamic tract [26]. Accordingly, changes in the spinothalamic tract are linked with the appearance of CPSP symptoms in many patients after stroke.

Haroutounian and colleagues in 2018 suggested that the origin of pain in CPSP need not be from ectopic CNS activity but could be from the afferent signals that mediate CNS sensitization. The prospective study conducted among eight CPSP patients by blocking the sensory signals from the peripheral pain field using ultrasound-guided block using lidocaine reduced pain to half the intensity in half an hour. The study results elucidate pain impulses derived from the peripheral sensory inputs and may be misconceived as CNS origin, contrasting with the hypothesis that central pain may be emerging within the CNS [5]. Central sensitization is also found to play a crucial role in the fundamental mechanism of nociplastic pain by exhibiting symptoms of sensitization. Therefore, patients with predominant clinical features of central sensitization may be conferred as nociplastic pain. IASP 2021 has defined the clinical criteria for nociplastic pain with the following requirements: pain lasting for minimum 3 months, a pain that has more of regional distribution, pain reported that cannot be completely explained by neuropathic or nociceptive mechanism and express evoked pain hypersensitivity symptoms like heat or cold allodynia, static or dynamic allodynia within the region of pain. Hence, the possibility of nociplastic pain cannot be excluded because of the presence of neuropathic or nociceptive pain when they are not entirely accountable for the pain [30].

Brain injury, like hemorrhagic stroke, leads to CNS inflammation and induces changes in the glial cells present in the central nervous system [35]. Microglia, a subtype of glial cells, are present as default macrophages in CNS and would become activated whenever homeostasis is disturbed. This macrophage activation is found to be assisted by the presence of P2 purinergic receptors on the glial cells [36]. Activated microglia lead to the generation of inflammatory mediators in addition to their functional role in the eradication of invading organisms [37]. Shreds of evidence expound microglia with upregulated expression of the P2 receptor family subtype (P2 × 7), post hemorrhage in the VPL nucleus of the thalamus. The P2 × 7 receptor, mostly expressed in microglia, in turn, commences the specific release of interleukin-1β (IL-1β) in addition to cytokines [36] and triggers the glutamate release in the nearby regions of stroke [37]. The consequences were evident in the thalamic cingulate pathway with extensive neuronal firing. Neuronal hyperexcitability observed with central pain was modified upon the inhibition of P2 × 7 at the acute post-hemorrhage stage blocking the microglial proliferation and proinflammatory cytokine release, eventually preventing CPSP. Additionally, microglia, as well as astrocytes, were activated by the highly expressed P2 receptor subtype P2 × 4 in a model of CPSP and steered in the release of the brain-related neurotrophic factor (BDNF) and cytokines mediated via the p38-mitogen-activated protein kinase phosphorylation [38]. Enhanced binding of BDNF to the tropomyosin receptor kinase B (TrkB) receptors activates the Na-K-CL cotransporter NKCLL and inhibits K-CL cotransporter KCC2 [37,39], interferes with CL^−^ concentration across the membrane, which turns down the hyperpolarizing effects of GABA and also directs to the disinhibition of the medial thalamus mediated from the inhibitory signals from the lateral thalamus. On treating CPSP rats with TrkB-Fc, the BDNF blocker drug manifested a reduction in a neuronal burst in the medial thalamus and attenuated mechanical allodynia. Therefore, the influence of inflammatory mediators contributing to neuropathic pain is implicated in CPSP [37]. Moreover, microglia–astrocyte activation triggered after thalamic hemorrhage can lead to a release of chemokine, SDF1 (stromal cell-derived factor 1), which on binding to its receptor CXCR4 plays a pivotal role in the development of CPSP through (HIF-1 α) hypoxia-inducible factor—1 α, thereby leading to positive feedback signals within glial cells–neurons and glial–glial cells and results in the generation of inflammatory mediators like IL-1β, IL-6, and TNF- α. CPSP induced by ITC (intra thalamic collagenase). SDF1 injections were found to be reversed or inhibited with the administration of the selective CXCR4 inhibitor, AMD 3100, or by microglial or astrocytic inhibitors. The study, in addition, reported the blockade of inflammatory mediators like IL-1β, IL-6, and TNF- α by AMD 3100 [40]. TNF- α formation in thalamic microglia mediated through NF-KB as well as ERK1/2 signaling is prompted with an increased expression of a non-receptor tyrosine kinase, Fgr belonging to the Src family following hemorrhage at the thalamus, thus inducing long-lasting hypersensitivity of pain responses including hyperalgesia (heat and cold) and mechanical allodynia contralaterally [41]. Microglial activation and proliferation are reported to be mediated via purinergic receptor (P2X) signaling, SDF1–CXCR4, and Fgr through NF-KB–ERK1/2 signaling and thereby contributing to the development of CPSP [42].

The binding of opioid peptides like enkephalins and dynorphins onto the opioid receptors (OR) has been indicated in the attenuation of pain response and may be involved in the pathogenesis of CPSP. Opioid receptors are widely expressed in the primary afferent neurons as well as post-synaptic neuron dendrites, and there is a release of opioids in the interneurons of the dorsal horn [25]. Research incorporating radiolabeled ligand ([^11^C] diprenorphine) to analyze the OR binding was conducted on 5 chronic CPSP patients and 12 healthy subjects. The outcome of the study displayed reduced OR ligand binding on significant pain-processing regions in the thalamus and different cortical regions among CPSP patients. In addition, former PET studies conducted on CPSP subjects have shown an augmented activity in most of these structures. The study findings aid in correlating the reports that stated therapeutic response failure after intake of opioids in CPSP patients, though opioids have been used in various neuropathic conditions due to their property to increase the threshold of pain sensitization. Furthermore, it supports a better understanding of the neurochemical properties of the structures in the pain circuit, though it could not derive the entire role of opioids in CPSP. Thus, further research is needed to reveal the perplexed role of opioids in the pathophysiology of CPSP [26].

## 6. Management of CPSP

**Figure 2:**
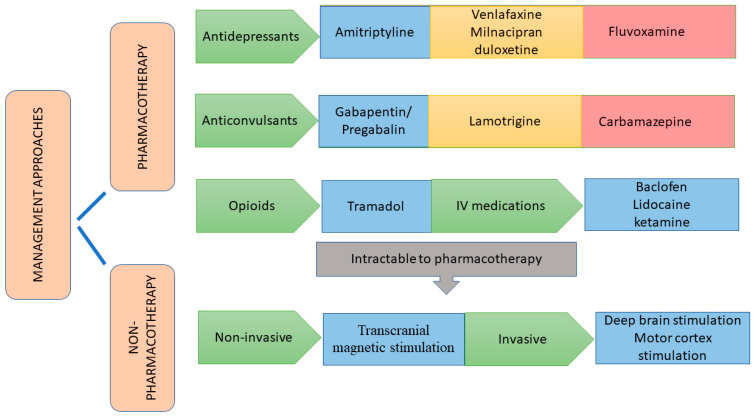
Management approaches to central post-stroke pain.

CPSP is a chronic state of pain where the treatment seems complex due to its variable clinical symptoms, moderate therapeutic efficacy, and limiting doses due to side effects. Moreover, there are limited randomized controlled studies on CPSP-centered therapy. Common management strategies incorporated in the CPSP treatment comprising pharmacotherapeutic and non-pharmacotherapeutic approaches have been discussed in Figure 2.

### 6.1. Pharmacotherapeutic Approaches

#### 6.1.1. Antidepressants

Various literature supports the tricyclic antidepressant (TCA) amitriptyline as the first-line treatment for CPSP while administered at a dose of 75 mg per day. Placebo-controlled crossover trials with amitriptyline demonstrated efficacy in treating CPSP. Though not all subjects responded to it, responders improved their condition within a month of the trial. Anticholinergic effects of TCA included dry mouth, constipation, and urinary retention, apart from cardiac side effects like orthostatic hypotension and cardiac arrhythmia. Hence, the use of TCA after stroke in elderly patients should be thoroughly monitored [43]. Other tricyclic antidepressants (TCAs) like nortriptyline are known to treat neuropathic pain, but their efficacy in patients with CPSP is unknown. The selective serotonin reuptake inhibitor (SSRI), fluvoxamine, was shown to be effective in CPSP patients at doses up to 125 mg daily who had a stroke within a year. The effectiveness of pain treatment appeared to be independent of the effect on depression in that study; however, no placebo-controlled studies have been conducted to confirm their efficacy for CPSP. Additionally, selective serotonin and norepinephrine reuptake inhibitors (SNRIs) show proven efficacy with a variety of chronic pain syndromes, but no studies have been conducted yet, specifically on CPSP. Venlafaxine, milnacipran, and duloxetine could be more effective than SSRIs in pain reduction with fewer side effects than TCAs [44].

#### 6.1.2. Anticonvulsants

Anticonvulsants are considerably the potential drug of choice in treating neuropathic pain syndromes. Among the group, gabapentin and pregabalin are considered second-line drugs to treat CPSP, but only a few controlled clinical trials have been conducted. Gabapentin, taken at a dose of 900 mg/day for consecutive 3 days and moderately increased up to 2400 mg/day, was found to attenuate the continuous pain in central neuropathic conditions, including CPSP, apart from diabetic peripheral neuropathy. In addition, pregabalin studied on a double-blinded placebo trial, with a dose of 150–900 mg/day, showed a remarkable alleviation in pain symptoms and complemented with improvement in anxiety and sleep quality. Pregabalin and gabapentin are reported with mild side effects like dizziness, nausea, and somnolence. In addition, it owes an enhanced safety profile. Therefore, it is considered an alternative drug of choice in the therapeutic management of neuropathic pain for patients who are unable to bear higher doses of TCA. Management of central pain by a single drug may not be efficient in providing optimum pain relief. Incorporating an antidepressant along with gabapentinoids as combination therapy provides added pain relief on secondary use of a reduced dose of each of the drugs, thereby also reducing side effects [43].

Lamotrigine studied on a randomized, double-blind, placebo-controlled trial comprising 27 CPSP patients showed a reasonable result with doses up to 200 mg/day. In comparison with the control group, 44% of the patients expressed diminished pain responses that were statistically significant at the highest tested dose of 200 mg daily when the trial followed for 2 months. Except for the occurrence of a mild rash, lamotrigine was well tolerated. However, Stevens–Johnson syndrome and toxic epidermal necrolysis (TENS) are serious potential side effects of lamotrigine that must be addressed with the patient.

Carbamazepine and amitriptyline, compared in a placebo-controlled crossover study, presented better results for carbamazepine only at 3 weeks, whereas amitriptyline was significantly better in alleviating pain at 2, 3, and 4 weeks. It is beneficial, especially at higher plasma levels. Probable side effects reported with carbamazepine are ataxia, rashes, hyponatremia, bone marrow dysfunction, and hepatic dysfunction. The side-effects profile of carbamazepine, as well as its drug–drug interactions, limits the pharmacotherapy of CPSP [44].

#### 6.1.3. Opioid Analgesics

Tramadol has pronounced effectiveness in the management of neuropathic pain, mediating centrally, unlike other opioids that are clinically effective only at higher doses and lead to adverse effects. In addition, tramadol has been suggested by the European Federation of neurological society as one of the treatment options for different types of neuropathic pains. The efficacy of tramadol may extend from its SNRI property and can be used in treating various neuropathic pain conditions. It possesses a few major side effects like reduction in seizure thresholds, serotonin syndrome in combination with 5-HT_3_ drugs, and a confused state among elderly patients [43]. However, no dependence potential is observed at therapeutic doses, unlike other opioids like morphine [45].

#### 6.1.4. Miscellaneous Drugs

Baclofen administered intrathecally was reported to be efficacious in attenuating stroke-induced central pain with minimal side effects. A double-blind, placebo-controlled crossover study administering lidocaine (5 mg/kg; 30 min) via the parenteral route in patients (*n* = 16) suffering from pain either induced by stroke (*n* = 6) or spinal cord injury (*n* = 10), significantly reduced the pain symptoms in 10 out of 16 patients [43]. The non-competitive NMDA blocker drug ketamine was also recorded for a reduction in pain scores among subjects suffering from central pain induced by spinal cord injury. Nevertheless, all the intravenously (IV) administered drugs studied were able to produce pain relief for a short period besides posing harmful side effects [46]. IV medications could thus be considered for administration as an ultimate step in a hospital setting, provided the pain does not respond to other therapeutic agents.

### 6.2. Non-Pharmacotherapeutic Approaches

Different experimental modalities can be accounted for in CPSP cases intractable to pharmacotherapy and may include repetitive transcranial magnetic stimulation (rTMS), invasive motor cortex simulation (MCS), deep brain stimulation (DBS), and exercise therapies [6]. rTMS is based on generating a high-intensity magnetic field at the lower regions of the brain by placing a magnetic coil tangential to the scalp of the patient’s head. It is a non-invasive method constituting a magnetic field that could induce an electric field activating the neurons directly. It is a safe method where the magnetic coil is placed over the cerebral cortex. The method has demonstrated positive effects on neuropathic pain treatment and could be related to the activation of presumably inhibitory projections from the motor and premotor cortices to sensory thalamic nuclei and from there to deeper structures like the brainstem or limbic system. A former study that consisted of neuropathic patients (*n* = 48), including CPSP (*n* = 24), expressed pain reduction compared to the control group when subjected to rTMS. Extensive literature addressing the beneficial effects of rTMS on CPSP patients is available as well [44]. Recurrent sessions of rTMS on the primary cortex increase the period of pain relief up to 2 to 3 weeks, notably devoid of severe side effects [47].

MCS has been tested in patients with neuropathic pain syndromes of various causes using implanted prefrontal epidural electrodes [44]. This invasive procedure inhibits the thalamus. In addition, it also modulates insular and cingulate cortical regions, thereby modifying pain pathways related to emotional components. The results of a study involving MCS unveiled that effective pain relief by the method could be achieved only with an intact spinothalamic cortical pathway [6]. A previous systematic review with data from 327 patients, with 193 CPSP patients, when subjected to MCS, suggested a positive response at a rate of 64%, where the response rate declined at the follow-up 55% [45]. However, MCS was not successful in treating CPSP subjects with severe motor weakness. Apparently, the precise location of MCS and accurate programming stimulation specifications are directly linked to the success of MCS [6,29].

DBS is a neurosurgical method that involves inserting deep stimulating electrodes into target brain regions through burr holes that, in turn, become linked to a pulse generator and will aid in controlling amplitude, pulse frequency, and contour [44]. DBS is a method of choice employed in the treatment of neurological disorders and refractory pain syndromes where the target structures are confined to periaqueductal or peri-third ventricular grey matter, as well as the sensory thalamus. The mechanism by which DBS relieves pain is not completely comprehended; nevertheless, two recently undergone trials involving DBS presented positive symptomatic relief that focussed affective cerebral sphere [48,49]. A previous study incorporating 45 CPSP patients that employed DBS treatment received optimistic results in controlling pain among 53%, which could be considered a progress indication for permanent implantation. In addition, patients with permanent implantation continued the pain alleviation even at the time of follow-up [44].

Physical therapies like acupuncture and TENS (transcutaneous electrical nerve stimulation) have shown temporary relief in pain among subjects with CPSP. A previous randomized controlled study conducted among CPSP patients subjected to 3 weeks of acupuncture contributed a significant attenuation in pain assessed with a visual analog scale (VAS) score. However, the need to evaluate the efficacy in a larger population is inevitable [6]. Alternatively, incorporating mirror therapy has gained interest in healing motor impairments observed after stroke. Mirror therapy is based on superimposing the mirror reflection of unaffected limb movements on the affected limb. A recent case report presented by Corbetta and colleagues showed that the application of mirror therapy for two weeks in a patient with CPSP after thalamic-capsular stroke having burning pain and sensory loss in the left upper limb showed a reduction of 4.5 points in the VAS score for the trained upper left arm and also maintained the pain reduction at one-year follow-up. Motor recovery attained by mirror therapy may be explained by an inhibition of improper proprioceptive inputs via visual information or by inducing sensorimotor plasticity [22].

## 7. Emerging Targets

Though the magnitude of the signs and symptoms of CPSP seems to be large, the positive studies on the mechanisms are welcoming. Lu et al. found the increased expression of P2X4 in peri thalamic lesions following hemorrhage in a new rat model of CPSP by intra thalamic autologous blood (ITAB) directly into the posterior lateral thalamic nucleus. Inhibition of P2X4 showed a remarkable reduction in allodynia and presented microglia-expressed P2X4 as a new drug target in the therapy of CPSP. In their successive research using ITAB-induced CPSP, it was also observed that the model could incite endocytosis of GABAaR, elevated levels of TNF-ά, and mechanical hypersensitivity. The results showed that P2X4 suppression lowered TNF-ά levels and overturned central disinhibition in addition to impairment of mechanical allodynia. Thus, the team also unveiled the regulatory role of P2X4 and TNF-ά/TNFR1/ GABAaR in CPSP, and further studies on this signaling could be a new area of therapeutic interest [31]. The study by Chen and colleagues reported the anti-nociceptive potential of 14, 15-Epoxyeicosatrienoic acids (EETs) when injected in a low dose (100 ng) on aiming the thalamic VPL nucleus [50]. Earlier, another research also demonstrated the role of 14, 15-EETs in central pain transmission, while an increase in the latency of tail flick was observed in rats on microinjection of the compound into the ventrolateral periaqueductal gray of the brain [37].

EETs are found to be short-lived due to chemical degradation by enzyme-soluble epoxide hydrolase (sEH). Incorporating soluble epoxide hydrolase inhibitors (sEHI) can sustain the level of EETs, thereby prolonging the duration of action of EETs. There were reports of a decrease in lipopolysaccharide-elicited allodynia in rats upon topical application of sEHI and EETs exogenously. The research conducted by Wagner and fellows demonstrated mitigation in pain symptoms of diabetic neuropathy in the murine model on administrating sEHI trans-4-[4-(3-adamantan-1-yl-ureido)-cyclohex- yloxy]-benzoic acid (t-AUCB) [51]. Further detailed studies on EET’s role as a modulator in the neuropathic pain mechanisms can be considered in developing a new treatment strategy for neuropathic pain symptoms in CPSP. There was an increase in the expression of anti-inflammatory microglial phenotype (M2) in the hippocampus after the administration of sEHI 4-phenyl chalcone oxide (4-PCO) following a cardiac arrest and cardiopulmonary resuscitation (CA/CPR). Adhering to the previous evidence, it is evident that after stroke incidence, the presence of sEHI/EETs can turn on, expressing the restorative microglial phenotype (M2). Central neuroinflammation regulated with EETs through sEHI controls central neuropathic pain and holds the possibility of development as a future pharmacotherapy [37].

Chronic pain has been greatly attributed to the release of proinflammatory cytokines, especially tumor necrosis factor-ά (TNF-ά), which is strongly associated with neuropathic pain [52]. Stroke incidence leads to the generation of reactive oxygen species (ROS) capable of switching glial cells to an active state and mediating the release of cytokines like tumor necrosis factor-ά, IL-1β, and IL-6 along with BDNF. The inflammatory mediator cytokine augments the excitatory effects of glutamate, and BDNF induces a diminished GABA inhibitory effect. Furthermore, cytokines released stimulate glial cells, which sequentially release more cytokines from the injured site, facilitating immoderate central neuroinflammation and central sensitization. Extensive research in targeting these perspectives of inflammatory reactions in CNS after stroke may help decrease the neuronal hyperexcitability in CPSP [38].

Reports suggest that adenosine (A_2A_R) antagonist SCH5826 is capable of inhibiting the neurotoxic effects of glutamate, thereby offering remarkable neuroprotection in the early stage of postischemic injury. Moreover, studies have shown impairment in immune cell infiltration and neuroinflammation on A_2_ agonist CGS21680 treatment after a week of stroke, owing to minimized damage in the brain [53]. Modulation of the long-chain fatty acid receptor (GPR 40) is reported to release β-endorphin. Researchers have also discovered the presence of GPR 40 in mice. The study conducted in a mouse model of CPSP showed repression in the pain score on treatment with GPR40 agonist [26], thereby recommending another potential target that needs further research.

## 8. Conclusions

It is common to experience post-stroke pains after the incidence of a stroke. The effective management of CPSP relies on recognizing and characterizing it from coexisting poststroke pains. The interval in observing the symptoms of CPSP makes it unrecorded during a medical examination in the acute period of post-stroke. The differences in the study groups and diagnostic measures used make the prevalence of CPSP low among stroke patients and are reported with variance in results. Though the thalamus is widely studied among CPSP patients, research also discloses the association of lesions in extra thalamic sites. Apparently, understanding central pain mechanisms that correlate with the pathophysiology of CPSP may aid in the early identification of central neuropathic pain induced by stroke. Amitriptyline and gabapentinoids are considered first-line and second-line agents in the treatment of CPSP, respectively. Monotherapy in treating CPSP might not be sufficient to provide optimum results. The combination of antidepressants with gabapentinoids used in clinical practice is found to be more effective with reduced drug doses. Non-invasive approaches could be considered for patients refractory to pharmacotherapy. Therapeutic options in CPSP are limited, and there is an urge to focus on new drug targets to optimize efficacy along with minimizing side effects. The negative impact of CPSP on the psychological well-being of the patients may lead to anxiety, depression, and sleep disturbances. Also, the extent of recovery from the pain symptoms seems to be moderate. Hence, cognizance of CPSP unquestionably helps in creating awareness among healthcare providers for early prognosis and timely management of CPSP.

## Figures and Tables

**Table 1:** Diagnostic criteria for CPSP.

**Author**	**Diagnosis Criteria**
Ri S, 2022 [23]	Somatosensory examinations: Initiated on the basis of sensory disorder, thermal and tactile hypersensitivities, and the presence of pain on the contralateral half, followed by thalamic stroke; Abnormalities in cold and warm sensations, constant or intermittent pain that worsens by exposure to cold temperature or palpating the affected side; Proprioceptive sensation, the status of cranial nerves, balance, and speech must also be assessed. The affected area can be colder than others; Partial or complete aberrations in pinprick and temperature sensation are often observed with patients while their proprioception and vibration sensation remain intact; Neuroimaging tests: Electroencephalogram (EEG) and somatosensory-evoked potential (SEP) examination can be confirmed with the observed somatosensory symptoms.
Osama A et al., 2018 [19]	Excluding the general causes of pain; Somatosensory examinations: Location of pain restricted to one side of the body/face or presence of pain on one side of the body and pain on the face on the other side; Assessing the stroke history along with the appearance of neurological signs and symptoms and the time period of onset of pain at or after stroke; Predicting sensory signs (+ or −) in the painful area, presence of evoked pain, or sensory dysfunction in an area enclosing the sensory abnormality; Neuroimaging tests: Analyzing the neuroimaging reports of the brain like a computed tomography (CT) scan or magnetic resonance (MR) and correlating sensory findings to the location of lesions.
Choi-kwan et al., 2016 [24]	Somatosensory examinations: Presence of constant or intermittent pain in a body region that may show (+ or −) sensory signs; Hyperalgesia or allodynia is present in most patients; Pain descriptors are explained as electrical, burning, cold, painful, stinging, or pins and needles; Confirm the painful sensation is neither emerging due to the movement of a joint nor the presence of tenderness at the site of pain; VAS score should be ≥40 for patients describing pins and needles; Neuroimaging tests: Verification of a corresponding stroke lesion by MRI.

## Data Availability

Not applicable.
